# Patient perceptions and expectations of an anticoagulation service: a quantitative comparison study of clinic‐based testers and patient self‐testers

**DOI:** 10.1111/scs.12195

**Published:** 2015-02-16

**Authors:** Arthur G. Money, Julie Barnett, Jasna Kuljis, Debbie Duffin

**Affiliations:** ^1^ Department of Computer ScienceBrunel University LondonUxbridge, LondonUK, UB8 3PH; ^2^ Haemostasis and Thrombosis UnitNottingham University Hospitals NHS TrustQueen's Medical Centre CampusDerby RoadNottinghamNG7 2UH

**Keywords:** self‐care, self‐testing, self‐management, patient expectations, patient perceptions, service quality, having choice, making choice, technology‐assisted health care

## Abstract

**Background:**

Government initiatives see the provision of technology‐assisted self‐care as one of the key areas in which there is capacity for improving quality of care whilst reducing costs. However, levels of patient engagement in self‐testing and management (STM) remain low. Little emphasis has been placed on understanding the patients' perspectives of the reasons for this limited engagement. Typically, patient engagement in STM is achieved via the provision of patient education programmes, which aim to enable patients to make the changes necessary to become competent self‐carers. However, placing the onus to change on the individual patient is unrealistic. If levels of patient engagement are to be improved, patient needs and expectations of clinical services must be better understood and service provision must be adapted accordingly.

**Objective:**

Explore patient perceptions and expectations of clinical service provision and their views of having and making choices about care.

**Methods:**

Participants [N = 191, 103 patient self‐tester managers (PSTMs) and 87 clinic‐based testers (CBTs)] completed the SERVQUAL and ChQ instruments to capture perspectives on service quality and choice, respectively. A comparative statistical analysis explored the similarities and differences between PSTMs' and CBTs' responses.

**Results:**

Clinic‐based testers' perceptions of service quality were significantly more positive than PSTMs', as were their expectations of the ‘tangible’ aspects of service delivery. PSTMs' expectations of service quality were significantly higher than their perceptions. PSTMs attributed significantly more value to making choices compared with CBTs.

**Conclusions and recommendations:**

To close the gap between PSTMs expectations and perceptions of service quality and better cater for their choice preferences, service providers may benefit from taking into account the following practice considerations: maintain frequent, timely, personalised and direct interactions with PSTMs; prioritise investment in resources to facilitate patient/practitioner interaction over tangible facilities; ensure that PSTMs are given the opportunity to make choices about their care.

## Introduction

As a result of a rising demand for healthcare resources 1, 2, government initiatives see patient participation in the delivery of their own health care as one of the key areas that has capacity for improving quality of service provision whilst also reducing costs 3, 4. Technological advancements in recent years have resulted in an ever increasing range of medical devices being made available over the counter, enabling patients to engage in point of care self‐testing and management (STM) of a wide range of conditions 5. As a result, now more than ever, patients are becoming more involved in the delivery of their own care, which is resulting in a lower frequency of face‐to‐face consultations with healthcare practitioners and increased levels of care responsibility being taken on by individual patients 6, 7, 8. The increased focus on technology‐assisted STM may in part be motivated by the need to develop more efficient and/or less resource intensive healthcare services. However, there is a belief that STM can often be more effective than more traditional clinic‐based services 6. Some of the key benefits of STM include the convenience of not having to make the journey frequently into clinic, carrying out tests at more appropriate times, improved levels of knowledge relating to the condition, self‐efficacy and health status, but also importantly, patients are empowered to have and make choices about their own care and become more equal partners in their own healthcare provision 9, 10.

For some time now, best practice guidelines have suggested that a more patient‐centred approach to care should be adopted, which empowers patients to have greater independence, control and choice regarding the decisions that are made about their care wherever possible 11, 12, 13, 14. Increased engagement in STM offers a promising way to enact these guidelines; however, thus far, patients have not necessarily embraced this possibility 7, 15. A survey of 383 participants found that 77% of patients were either not willing or did not feel able to adopt new behaviours associated with STM; the remaining 23% that had engaged in STM believed they could maintain these changes when in crisis 16. Increasing uptake and ensuring that STM interventions are effective requires traditional patient–practitioner care paradigms to be redefined, placing the principles of patient centredness, collaboration, engagement and patient empowerment at the heart of this redefinition 17, 18. Furthermore, there is an urgent need to identify and cater for the needs of patients who engage in STM care models.

Until now, policymakers have placed relatively little emphasis on understanding patient perspectives and the reasons for limited patient engagement with this new care paradigm, but have rather focused on demonstrating the efficacy of this approach via numerous randomised control trials with patients who already engage in STM 19.

## Self‐testing and management for oral anticoagulation therapy

Oral anticoagulant therapy (OAT) is concerned with the management of irregularities in blood coagulation (i.e. the process in which blood clots from) and is often required as part of the treatment of chronic conditions where there is risk of harmful blood clots forming or growing larger. Examples include atrial fibrillation, coronary artery disease and individuals who are at risk of deep vein thrombosis, pulmonary embolism and stroke 20. Oral anticoagulant therapy has a narrow therapeutic index; therefore, to prevent adverse health effects of over‐ or undercoagulation, regular monitoring of anticoagulant therapy is required 21. Oral anticoagulants, such as warfarin, are prescription drugs that reduce the body's ability to form clots in the blood; in effect, they increase the amount of time it takes to form a clot, but do not dissolve clots that have already been formed. In 2005, it was estimated that more than 1.25 million people (2% of the total general practice population) in the United Kingdom were taking an oral anticoagulant 22. This figure has increased steadily since then 6. Portable coagulometers test the prothrombin time (PT) and the international normalised ratio (INR) of patients that take oral anticoagulants to reduce the time required for blood to clot 23. Prothrombin time is the time taken for patient blood to clot, and INR relates to the ratio of a patient's PT compared with a normal sample. These devices have enabled INR patient STM to become a reality for some 24. In the United Kingdom, there are typically two types of patients who utilise anticoagulation services:

1. Clinic‐based testers (CBTs): patients visit the anticoagulation clinic regularly for PT/INR testing, treatment and advice.
2. Patient self‐tester managers (PSTMs): patients use a portable coagulometer to regularly test their own PT/INR levels at home. They stay in contact with the anticoagulation clinic via the telephone and visit the clinic every 12 months in order to check the calibration of the device.

Existing INR STM research typically focuses either on the efficacy of existing interventions by comparing health‐related outcomes of INR STM with normal clinic‐based care, or on the benefits and challenges associated with INR STM as perceived by the patient and/or practitioner. A number of systematic reviews and meta‐analyses that consider health‐related outcomes have provided evidence that INR STM is an effective and safe intervention strategy compared with the traditional clinic‐based equivalent 6, 22, 24, 25, 26. In terms of health‐related outcomes, PSTMs have been found to spend more time within the therapeutic INR range 24 and experience less thromboembolic events 6, 25. As a result, PSTMs have been found to benefit from reduced levels of overall mortality and complications directly associated with being outside of the therapeutic range 25, 26.

Research has also identified the perceived benefits of INR STM from the patient and practitioner perspective. Shah and Robinson 7 conducted an analysis of discussion threads of PSTMs posted on online blogs to explore patient perceptions of INR STM. They found that benefits of STM include time saving, reduced necessity to travel, an increased level of choice and control of their condition, and more peace of mind as a consequence of being able to test when deemed necessary. Yang et al. 20 and Fitzmaurice et al. 22 found that patients value the greater level of independence and convenience that INR STM affords. Bloomfield et al. 25, Matchar et al. 24 and Braun et al. 27 all found higher levels of reported patient satisfaction and quality of life for PSTMs compared with CBTs. From an operational perspective, INR STM is seen to deliver improvements in resource allocation by freeing time for both physicians and laboratory personnel 7. However, it is still unclear how cost‐effective INR STM interventions are compared with traditional clinic‐based interventions 25.

## Patient perceptions and nonengagement with INR STM

As a consequence of increased efficiencies, improved health outcomes and the perceived therapeutic advantages associated with INR STM, for some time now, best practice guidelines have recommended that practitioners offer STM options to patients 12, 28. However, despite these recommendations, uptake of INR STM has been slow 6, 29. Connock 21 found that only 14% of patients who are eligible to adopt INR STM were actually willing to engage in this mode of care delivery.

A small number of INR‐specific studies have, to some extent, started to explore the reasons for this. Wittkowsky et al. 30 found that in the United States, the cost of purchasing the self‐testing device and the disposable test strips was a key reason for lack of patient engagement. It is unclear, however, whether this is still the case as the range of indications covered by private health insurers in the United States has been expanded since this study 31. In a Cochrane review which included 4723 participants, Garcia‐Alamino et al. 26 identified that the primary reason for low levels of engagement with the INR STM delivery care model was not, as previously thought, that healthcare providers were not offering patients the opportunity to become STMs, but rather that, despite patients often being given the opportunity to engage with the INR STM model, their preference was to continue with the clinic‐based mode of care they were already receiving. An inability of patients to complete training was also identified as a contributing factor. Young and Skorga 32 found that taking on the responsibility of one's own care can be perceived as a frightening prospect for some patients, and hence, may be a key factor that contributes to the low levels of engagement. Gardiner et al. 29, 33, 34 carried out a series of studies in the United Kingdom and found that patients felt reassured and well supported within the hospital anticoagulation clinic environment. They feared that if they opted for the INR STM option, they would lose the expert advice and the quality of service that are available to them as CBTs. Fear of reduced expert advice, support and quality of service as a result of engaging with STM do not seem to be unique to the INR domain. Jordan and Osbourne 35, who considered the challenges associated with chronic disease self‐management education programmes, found that patients expressed concerns relating to the quality of service that they may receive if they were to embrace the STM care delivery model. In an ethnographic patient‐focused study, Keilman et al. 36 identified issues relating to patients experiencing feelings of abandonment by healthcare professionals as a result of engaging in STM.

## Common approaches to achieving patient engagement with STM

A common approach to achieving patient engagement with STM is to focus on providing education with the aim of enabling them to make the necessary changes to become expert patients who are managers of their own health condition 37, 38. The primary tool used to achieve these changes to date has been via the provision of STM patient training programmes 39, 40. These programmes aim to educate patients to ensure that they change their behaviour and develop the appropriate knowledge that is deemed necessary to enable initial and ongoing engagement with the STM care model 39. In addition to having specialist knowledge of a particular chronic condition and its management, skills that patients are expected to adopt as a result of attending STM training programmes include the following: (i) being able to make choices and actively participate in decision‐making with health professionals; (ii) negotiating a self‐management care plan with health professionals; and (iii) having the confidence and ability to access and use clinical health services effectively 41. There is an expectation that patients will successfully achieve changes in their attitude, behaviour and skills set as a direct consequence of engaging in these training programmes and consequently engage effectively with the STM care models clinical services that are available to them 42. Some criticisms of STM patient training programmes, however, have been that their content focuses on changing patient behaviour to make the most of the existing services that are available to them, hence minimising the requirement to adapt and improve clinical services to accommodate the needs of the patient 38. Furthermore, some researchers have observed that the content of STM support programmes is often designed according to the underpinning principles of the traditional medical model, the effectiveness of which is evaluated according to empirical and societal focused health outcomes as opposed to health outcomes defined by the patient 43. Hence, the extent to which these initiatives truly empower patients to achieve effective self‐management is questionable 19, 44.

Placing the onus to change on the individual patient, rather than making changes to the broader context in which the care is provided, is unrealistic and may be a key factor that constrains patient engagement with STM 36, 45. Indeed, this issue has been highlighted by The Scottish Executive Health Department 46 who stated, ‘for too long people have been made to fit the services rather than services being made to fit the people’. If the low level of uptake of STM is to be addressed fully and improved, there is a need to consider how society, healthcare policy, healthcare services, infrastructure and healthcare professionals can be changed to support the needs of STM patients 47. The notion of ‘whole system change’ has been said to be paramount to the success of the patient‐centred care agenda and is necessary if individual's capacity for engagement in STM practice is to be optimised 45. There is a particular need for health services to better understand the needs, expectations and perceptions of the patient in their new role as self‐carers and take account of these understandings to design systems and technologies that support them in their new expert patient roles 36. Whole system change to reflect the needs and perceptions of STM patients does not only include changing professional attitudes, but also includes changing the resources and services that are made available to patients in order to better reflect and accommodate their needs 48.

Little attention has been paid to gaining insights into what STM patient needs and expectations of clinical services are, and identifying what contextual adaptations can be made to clinical services in order to create the appropriate preconditions that enable patients to more readily engage with their role as an expert patient 19, 45. If levels of patient engagement in STM are to be improved, it is crucial that patient needs and expectations of clinical services are identified and service provision is adapted to accommodate these needs 49. This is particularly appropriate, when considering that fear of reduced service quality has been identified as a key factor which contributes to nonengagement in STM in practice.

## Service quality and patient engagement

Quality of service within the healthcare context is defined as the extent to which patients are satisfied with the service they are receiving, and is considered to be a key indicator in effective healthcare provision 50. Maximising service quality provision to patients is important as it has been found to be closely and positively correlated with measures of patient satisfaction within the healthcare context 51. Measures of service quality have also been shown to be positively correlated with patient self‐efficacy, adherence to treatment programmes and overall health outcomes 52, 53. There is also evidence to suggest that improved service quality impacts positively on patient uptake and engagement in STM practices 52, 53, 54, 55.

Identifying appropriate service quality measures, and the criteria which could be taken into account to comprehensively and effectively evaluate health service quality, is an ongoing research topic in its own right 55. Compared with the evaluation of the quality of tangible goods, service quality is inherently more difficult to define and measure, due to its intangibility 56. Moreover, definitions of health service quality may vary depending on the stakeholder's perspective and their role within service delivery. For example, Mosadeghrad 57 identified nine types of stakeholder typically involved in health service delivery: patients, relatives, providers, managers, policymakers, payers, accreditation staff, suppliers. As a result of carrying out interviews and focus groups with over 700 individuals, it was concluded that each of these respective stakeholder groups defines health service quality differently. This highlights the fact that defining the full range of discrete criteria that specific types of stakeholder consider to be important in health service quality evaluation poses significant challenges. Similar to the definitions of health service quality being a function of stakeholder type, definitions of health service quality may also vary depending on the type of service which is being delivered and indeed the types of health conditions which are being treated 58, 59. Another way of considering the range of criteria which may be used to measure health service quality is to view criteria as being a function of one of two distinct categories: (i) internal/supply‐side measures of quality; and (ii) external/demand‐side measures of quality 60. The former focuses on criteria which are considered as important from the healthcare provider's perspective and may include criteria, such as the extent to which services adhere to quality standards and guidelines, whether the expected clinical outcomes are being achieved, the extent to which service provision adheres to predetermined clinical pathways, the efficiency of care provided. The latter often focuses specifically on the patient's experience of engaging with a service and takes into account the extent to which the service provided meets the needs and expectations of the patient. Although external/demand‐side measures typically exclude criteria which are often associated with measures of quality, such as health outcomes, the strength of such measures is that they focus on process and the customer/patient perceptions of a service and measure the extent to which patient expectations and needs are being met. Meeting patient expectations and needs is considered to crucially determine whether patients actually choose to use a service in the first instance, and hence has a direct impact on levels of patient engagement and the effectiveness of health service delivery more generally 55, 59.

Despite the often context‐specific nature of service quality definitions, existing literature within the service quality domain proposes that there are some common and well‐founded external/demand‐side dimensions which span across a range of stakeholders and service delivery contexts 55, 61. A widely accepted view in demand‐side service quality evaluation is that it is important to make a distinction between patient expectations and their actual experiences of a service 50. Sofaer and Firminger 62 emphasise the importance of making this distinction and highlight that it is crucial to conceptualising measurement of service quality along these two distinct perceptual standpoints. Therefore, a common approach to evaluating service quality, and identification of the features of a service that may benefit from improvement, is to measure the gap that exists between patient expectations and perceptions of how the service is actually being delivered 63. A number of quality and satisfaction measures take this approach to evaluating service quality in health care; the most commonly of which is SERVQUAL 49, 53, 64. The SERVQUAL instrument is considered to be the most comprehensive, useful and widely used instrument for measuring service quality from a service user perspective 59. SERVQUAL uses five core dimensions of service quality to evaluate whether there are differences between expectations and perceptions of these five dimensions, which are tangibles, reliability, responsiveness, assurance and empathy. A detailed description of the SERVQUAL instrument used in this study is provided in the next section, which includes all of the questions that make up the SERVQUAL instrument.

## The value of choice

Enabling patients to make choices about their own care is considered to be a central component to improving health 65. Choice is also seen as a key component in the shift towards STM, patient empowerment, patient centredness and patient–practitioner shared decision‐making 66, 67, 68. There is increasing recognition that having an understanding the patients who prefer to be offered choices and those who prefer to take a more passive role in decision‐making is more likely to ensure that patient needs are better accommodated and catered for 69. However, research that considers choice within the healthcare context is limited. The few studies that have been carried out have discovered that choice is not a unidimensional construct and that it may be more usefully considered as relating to two aspects of choice: ‘having choices’ and ‘making choices’ 66. In general, most patients value having choices which they perceive as being central to maintaining autonomy and enhancing self‐efficacy. However, patients may value making choices to a lesser extent, feeling that liaison with and even reliance on expert health professionals is the ideal scenario here 67. This study also found that there was a significant positive correlation between having and making choices, and a greater endorsement of the value of both constructs was related to higher educational status. These findings may offer some explanation of the findings presented in the previous section, which suggested that some of the reasons for nonengagement with INR STM care delivery were as a result of patients' fear of losing expert advice and experiencing feelings of abandonment and perhaps fearing that they will be center to make choices on their own when opting for the STM care delivery models. It is thus worth examining whether profiles of patient choice preferences may vary as a function of the type of care they currently engage with, that is clinic‐based testing or STM. If this were found to be the case, it would provide valuable insights into the extent to which patients may value the opportunity to have and make choices about their care.

The remainder of this paper is structured as follows. The next section outlines the details of an anticoagulation clinic study carried out with CBTs and PSTMs to explore perceptions and expectations of service quality and their respective views of having and making choices. The results of this study are then presented. The implications of the findings in the context of existing research are then discussed before drawing conclusions.

## This study

In response to the need to better understand how clinical service provision can be adapted to better accommodate the needs of PSTM patients, this study explores patient perceptions and expectations of service quality and whether these vary for patients who are managing their condition within two models of health care: CBTs and PSTMs. Furthermore, this study aimed to gain better understanding of the extent to which each of these two distinct patient groups values having and making choices. Participants completed a service quality questionnaire which asked them to report on their perceptions and expectations of the service they receive as patients of the same anticoagulation clinic. CBTs and PSTMs were also asked to report on the extent to which they value having and making choices relating to their care. Analysis of questionnaire responses served as means of developing a profile of the service quality and choice factors that PSTM patients expect and value most compared with CBTs, and hence should be delivered if patient engagement in STM practice is to be optimised. Specifically, the following research questions are addressed in this study:

RQ1. What are patients' expectations and perceptions of anticoagulation clinic service quality, and how do these differ *between* CBT and PSTM patient groups?

RQ2. What are patients' expectations and perceptions of anticoagulation clinic services, and how do these differ *within* CBT and PSTM patient groups?

RQ3. What are the similarities and differences between CBT and PSTM patient groups with regard to the way in which they value having and making choices?

### The anticoagulation clinic and existing contract of care

The anticoagulation service considered in this study has approximately 180 individuals enrolled as PSTMs, which is one of the largest PSTM cohorts associated with one anticoagulation clinic in the United Kingdom. This number is still a small fraction of the total number of CBTs enrolled at this clinic which is in excess of 7000. The anticoagulation clinic is situated within a city hospital, which is located in the north of England and currently employs in excess of 6000 staff.

In the first instance, patients join the anticoagulation clinic as CBTs. After a period of regular face‐to‐face consultations (typically twice weekly) to stabilise INR readings and gain some insight into how the patient is coping with the new condition, clinical staff may offer the patient the opportunity to become a PSTM. One essential criterion that must be met in order to become a self‐tester is that the patient must have the dexterity to carry out the test themselves, or have a relative who is willing to assist them carrying out the test. If the patient is offered the opportunity to become a self‐tester, they have the choice of accepting or rejecting this as an option. PSTMs typically visit the clinic in person once every 12 months, with the key aim of having their coagulometer calibrated, so that it provides accurate readings for the coming year. The contract of care, however, clearly states that PSTMs are expected to report their INR test results to the clinic, in the form of a recorded telephone message, at agreed dates throughout the year. Once the reading is received, the anticoagulation clinic sends, by post, a recommended dosing profile to the PSTM along with the next date they are expected to share their INR test results with the clinic. In addition to the telephone message service, PSTMs are provided with a telephone contact number which enables them to speak with an anticoagulation specialist if they wish. PSTMs are also able to request a face‐to‐face meeting with a member of clinical staff, and likewise, clinical staff reserve the right to request a face‐to‐face meeting if it is deemed necessary.

Clinic‐based testers maintain more regular face‐to‐face contact and receive treatment advice directly from the clinic‐based staff as a result of regular visits to the anticoagulation clinic. The frequency of visits is determined by the stability of their INR results and the clinical judgement of the practitioner. Patients are seen on an appointment basis, and dosing profiles are presented to the patient at the end of the face‐to‐face testing session along with the date of their next appointment. CBTs also have access to the telephone service which enables them to speak with a member of clinical staff in between face‐to‐face appointments if they so wish.

### Survey instruments: measuring service quality and choice

This study used the SERVQUAL instrument to capture patient expectations and perceptions of the anticoagulation service quality. SERVQUAL is a well‐established instrument used for measuring service quality 63. The essence of SERVQUAL is to measure the gap that exists between the expected levels of service quality (expectations) and those actually perceived (perceptions) by existing users of a service. This instrument has been specifically adapted for use in health care 49 and is made up of 19 matching statements each of which appears twice (38 statements in total): once to measure expectations of the service and again to measure service perceptions. The 19 statements are considered along five main dimensions/subscales: tangibles, reliability, responsiveness, assurance and empathy 50, 70. The SERVQUAL dimensions are defined as follows 71:

1. Tangibles: Appearance of physical facilities, equipment, personnel and communication materials.
2. Reliability: Ability to perform the promised service dependably and accurately.
3. Responsiveness: Willingness to help customers and provide prompt service.
4. Assurance: Knowledge and courtesy of employees and their ability to convey trust and confidence.
5. Empathy: The caring, individualised attention the organisation provides its customers.

**Figure 1:**
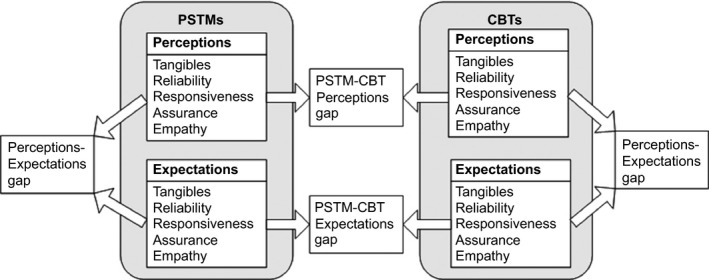
SERVQUAL expectations and perceptions comparisons.

The SERVQUAL measures enable a within‐group evaluation of whether a service is delivering service quality below or in excess of service users' expectations. Furthermore, when two or more groups of service users complete the SERVQUAL instrument, a comparison can be carried out to characterise any differences in their perceptions and expectations. In the case of this study, SERVQUAL enabled a within‐ and between‐groups comparison of CBTs and PSTMs perceptions and expectations of the anticoagulation service. These comparisons enable identification of how CBTs and PSTMs expectations and perceptions, of the same anticoagulation service, vary in terms of service quality dimensions and provide valuable insights into which aspects of a service should be adapted to cater for PSTMs needs and thus facilitate increase uptake and engagement in STM practice. Figure 1 shows the main SERVQUAL dimensions and how these relate to within‐ and between‐groups comparison of expectations and perceptions of an anticoagulation service.

All participants were also asked to complete the ChQ instrument 66, which consists of eight statements in total, four of which relate to having choices and the remaining four to making choices. All items on the SERVQUAL instrument and the ChQ instrument were ranked according to a seven‐point Likert‐type scale as ‘strongly agree' (7), ‘moderately agree’ (6), ‘slightly agree’ (5), ‘neutral’ (4), ‘slightly disagree’ (3), ‘moderately disagree’ (2) and ‘strongly disagree’ (1).

In addition to completing the SERVQUAL and ChQ instruments, all participants were asked to provide demographic information and were also asked to rate their satisfaction with the following: (i) the way in which they receive their INR results; (ii) the discussions they have with the anticoagulation clinic about INR‐related issues; (iii) warfarin as an anticoagulant. Participants selected answers from a five‐point Likert‐type scale: ‘very satisfied’ (5), ‘fairly satisfied’ (4), ‘neutral’ (3), ‘fairly unsatisfied’ (2) or ‘very unsatisfied’ (1).

### Participant recruitment

Participants were recruited from an NHS anticoagulation service located in England, United Kingdom. Both PSTMs and CBTs were recruited from the same service. PSTMs were invited to complete the questionnaire via postal invitation and clinic reception staff invited CBTs to complete the questionnaire when visiting the clinic. A follow‐up letter was sent to PSTMs 8 weeks after the initial invitation was sent if a completed questionnaire had not yet been received. Inclusion criteria were patients over the age of 18 who were current users of the anticoagulation service as either PSTMs or CBTs.

### Ethics approval

The study was reviewed and approved by Brunel University Research Ethics Committee prior to review and approval by the NHS London City and East Research Ethics Committee and the hospital in question's Integrated Clinical Research Centre. All participants taking part in this research were guaranteed confidentiality and anonymity. Participants were informed of their right to withdraw from the study at any time.

### Data analysis

IBM SPSS Statistics Package Version 18 was used to analyse the survey responses collected for this study. Descriptive statistical analysis was carried out on the demographic data. The Cronbach's alpha test for internal validity was applied to each of the five SERVQUAL constructs and the two ChQ constructs. Cronbach's α values around 0.6 and higher were considered as indicating an acceptable level of reliability, particularly given that all constructs were made up of relatively small numbers of items 72. For the SERVQUAL responses, the differences between PSTMs and CBTs expectations and perceptions of the anticoagulation service were assessed with independent samples and paired samples *t*‐tests for each of the five constructs. Analysis of the ChQ responses involved independent samples *t*‐tests to compare PSTMs and CBTs responses. Pearson's correlation coefficient was used to characterise the nature of relationship between having and making choices for each patient group.

## Results

A total of 340 patients were invited to complete the survey, 170 PSTMs and 170 CBTs. Of these, a total of 104 PSTMs (61%) and 87 CBTs (51%) completed the survey. A demographic characterisation of the sample is presented in Table 1.

**Table 1:** Demographics and INR testing activity data

	ST	ST %	CBT	CBT %	Overall	Overall %
Gender						
Male	62	59.62	43	49.43	105	54.97
Female	38	36.54	44	50.57	82	42.93
Not reported	4	3.85	0	0.00	4	2.09
Age						
18–25	2	1.92	1	1.15	3	1.57
26–40	11	10.58	6	6.90	17	8.90
41–55	12	11.54	16	18.39	28	14.66
56–70	47	45.19	40	45.98	87	45.55
71–80	23	22.12	20	22.99	43	22.51
Over 80	5	4.81	4	4.60	9	4.71
Not reported	4	3.85	0	0.00	4	2.09
Education						
Primary	2	1.92	2	2.30	4	2.09
Secondary	16	15.38	17	19.54	33	17.28
University	18	17.31	7	8.05	25	13.09
Postgraduate	47	45.19	47	54.02	94	49.21
Other	12	11.54	8	9.20	20	10.47
Not reported	9	8.65	6	6.90	15	7.85

Regarding the gender of participants that took part in this study, 59.2% of PSTMs were male and 36.54% were female whilst 3.85% did not report their gender. CBTs that took part in the study were 49.43% male and 50.57% female. The largest proportion of PSTMs and CBTs were aged between 56 and 70 years old with totals of 45.19 and 45.98%, respectively. In both samples, the smallest proportion of participants were aged between 18 and 25, 1.92% were PSTMs and 2.30% were CBTs.

### Perceptions and expectations of anticoagulation clinics

First we explored patients' expectations and perceptions of an anticoagulation clinic and the extent to which these differ between CBT and PSTM patient groups. This was achieved by considering the discrete aspects of service quality included in the SERVQUAL instrument. The differences between PSTM and CBT perceptions and expectations were compared, respectively, the results of which are presented in Tables 2 and 3. With regard to expectations, the Cronbach's alpha scores for assurance for both PSTMs and CBTs did not attain acceptable levels of reliability 72 and therefore will not be considered further. Item P10 for CBTs was deleted from SERVQUAL responses, so that the Cronbach's alpha score for responsiveness met the necessary reliability threshold. The corresponding item P10 was also deleted from PSTM responses, so that a like‐for‐like comparison could be made between CBT and PSTM responsiveness constructs.

**Table 2:** PSTM and CBT perceptions comparison

Construct	Perceptions Items: Response options: (1)Strongly disagree to (7)Strongly agree	Cronbach's alpha		Mean (PSTM)	Mean (CBT)	Gap score (PSTM–CBT)	Sig. (two‐tail)
		PSTM	CBT				
Tangibles	P1. My anticoagulation clinic has up‐to‐date equipment	0.81	0.75	5.92	6.31	−0.38	0.002^a^
	P2. The physical facilities in my anticoagulation clinic are visually appealing						
	P3. The printed materials for my anticoagulation clinic are visually appealing						
	P4. Staff in my anticoagulation clinic are neat in appearance						
Reliability	P5. When patients have problems, staff in my anticoagulation clinic are sympathetic and reassuring	0.82	0.82	6.28	6.64	−0.36	0.001^a^
	P6. My anticoagulation clinic provides its services at the time they promise						
	P7. Staff in my anticoagulation clinic tell patients exactly when services are performed						
	P8. My anticoagulation clinic insists on error‐free records						
	P9. Staff in my anticoagulation clinic have a sincere interest in solving patients' problems						
Responsiveness	P10. Staff in my anticoagulation clinic provide prompt service	0.73 (Item P10 deleted)	0.71 (Item P10 deleted)	6.37	6.88	−0.51	0.000^a^
	P11. Staff in my anticoagulation clinic are always willing to help patients						
	P12. Staff in my anticoagulation clinic are never too busy to respond to patients' questions						
Assurance	P13. Staff in my anticoagulation clinic instil confidence in patients	0.79	0.73	6.44	6.85	−0.41	0.000^a^
	P14. Staff in my anticoagulation clinic are polite						
	P15. Staff in my anticoagulation clinic have the knowledge to answer patients' questions						
Empathy	P16. My anticoagulation clinic has hours convenient to all patients	0.80	0.60	6.07	6.63	−0.56	0.000^a^
	P17. Staff in my anticoagulation clinic give patients personal attention						
	P18. Staff in my anticoagulation clinic have patients' best interests at heart						
	P19. Staff in my anticoagulation clinic understand the individual needs of patients						

Significant at 95th percentile

a Significant at 99th percentile

**Table 3:** PSTM and CBT expectations comparison

Construct	Expectations items: Response options: (1) Strongly disagree to (7) Strongly agree	Cronbach's alpha		Mean (PSTM)	Mean (CBT)	Gap score (PSTM–CBT)	Sig. (two‐tail)
		PSTM	CBT				
Tangibles	E1. An excellent anticoagulation clinic will have up‐to‐date equipment	0.68	0.78	6.15	6.39	−0.25	0.023^a^
	E2. The physical facilities in an excellent anticoagulation clinic will be visually appealing						
	E3. The printed materials for an excellent anticoagulation clinic will be visually appealing						
	E4. Staff in an excellent anticoagulation clinic will be neat in appearance						
Reliability	E5. When patients have problems, staff in an excellent anticoagulation clinic will be sympathetic and reassuring	0.82	0.78	6.72	6.73	−0.01	0.883
	E6. An excellent anticoagulation clinic will provide its services at the time they promised						
	E7. Staff in an excellent anticoagulation clinic will tell patients exactly when services will be performed						
	E8. An excellent anticoagulation clinic will insist on error‐free records						
	E9. Staff in an excellent anticoagulation clinic will have a sincere interest in solving patients' problems						
Responsiveness	E10. Staff in an excellent anticoagulation clinic will provide prompt service	0.70	0.63	6.60	6.68	−0.07	0.367
	E11. Staff in an excellent anticoagulation clinic will always be willing to help patients						
	E12. Staff in an excellent anticoagulation clinic will never be too busy to respond to patients' questions						
Assurance	E13. Staff in an excellent anticoagulation clinic will instil confidence in patients	–	–	–	–	–	–
	E14. Staff in an excellent anticoagulation clinic will be polite						
	E15. Staff in an excellent anticoagulation clinic will have the knowledge to answer patients' questions						
	E16. An excellent anticoagulation clinic will have hours convenient to all patients						
Empathy	E17. Staff in an excellent anticoagulation clinic will give patients personal attention	0.72	0.58	6.62	6.68	−0.06	0.402
	E18. Staff in an excellent anticoagulation clinic will have patients' best interests at heart						
	E19. Staff in an excellent anticoagulation clinic will understand the individual needs of patients						

a Significant at 95th percentile

The between‐groups comparison of perceptions of the service they currently receive, presented in Table 2, revealed that both groups generally agreed with the survey statements. All mean values were above six with the exception of the mean PSTM score for tangibles which was 5.92. This indicates that overall, both PSTMs and CBTs tended to agree with the positive statements that their anticoagulation clinic currently delivers positively on all five service quality constructs. However, there were significant differences between the groups in that CBTs rated the anticoagulation service significantly more positively than PSTMs (indicated by the negative gap scores) for all five constructs: tangibles (p = 0.002), reliability (p = 0.001), responsiveness (p = 0.000), assurance (p = 0.000) and empathy (p = 0.000). Given that both PSTMs and CBTs were reporting on the same anticoagulation clinic, it would seem that there are key differences in the way that the existing service is perceived by these two patient groups, specifically relating to the service quality constructs measured by SERVQUAL.

The between‐subjects comparison of PSTM and CBT expectations presented in Table 3 reveals that both groups generally agreed with the survey statements and in all cases mean scores were in excess of six. In absolute terms, all mean scores were higher for CBTs than for PSTMs. The tangibles score was significantly higher for CBTs than PSTMs (p = 0.023) indicating that CBTs tend to have higher expectations of the tangibles within the anticoagulation clinic compared with PSTMs.

Overall, comparing the results presented in Tables 2 and 3, with the exception of tangibles, PSTMs and CBTs do not appear to have significantly different expectations of their anticoagulation service. However, despite higher expectations for tangibles and similar expectations for reliability, responsiveness and empathy, CBTs perceive the services they receive more favourably than PSTMs.

### Within‐groups perceptions and expectations

Next, we sought to explore what patient expectations and perceptions are of anticoagulation service quality and how these differ within the CBT and PSTM patient groups. This was explored by evaluating the service gaps that exist for PSTMs and CBTs and comparing the gap between the perceptions and expectations for each respective patient group. Table 4 presents the gap scores and significance of differences in these service evaluation scores for PSTMs. Table 5 presents a similar profile of results for CBTs. Negative gap scores indicate that perceptions of the service do not meet patient expectations and positive gap scores indicate that the service delivers service beyond patient expectations. Cronbach's alpha reliability scores for the assurance construct were not acceptable and hence this construct will not be considered further. Items P10 and E10 for CBTs were deleted from the SERVQUAL responses, so that the Cronbach's alpha score for CBT responsiveness met the necessary reliability threshold.

**Table 4:** PSTM perceptions and expectations comparison

PSTM perceptions–expectations						
Construct	Cronbach's alpha perceptions	Reliability expectations	Mean perceptions	Mean expectations	Gap score	Sig. (two‐tail)
Tangibles	0.81	0.68	5.92	6.15	−0.23	0.011^a^
Reliability	0.82	0.82	6.28	6.72	−0.45	0.000^a^
Responsiveness	0.78	0.70	6.32	6.60	−0.28	0.004^a^
Empathy	0.80	0.72	6.07	6.62	−0.57	0.000^a^

a Significant at 99th percentile

**Table 5:** CBT perceptions and expectations comparison

CBT perceptions–expectations						
Construct	Cronbach's alpha perceptions	Cronbach's alpha expectations	Mean perceptions	Mean expectations	Gap score	Sig. (two‐tail)
Tangibles	0.75	0.78	6.31	6.39	−0.09	0.245
Reliability	0.82	0.78	6.64	6.73	−0.09	0.125
Responsiveness	0.71 (P10 deleted)	0.61 (E10 deleted)	6.88	6.73	+0.15	0.016^a^
Empathy	0.60	0.58	6.63	6.68	−0.06	0.278

a Significant at 95th percentile

With regard to PSTMs (Table 4), for all four service quality constructs considered, there were significant negative gap scores for tangibles = −0.24 (p = 0.011), reliability = −0.48 (p = 0.000), responsiveness = −0.28 (p = 0.004) and empathy = −0.57 (p = 0.000) demonstrating that expectations exceeded perceptions.

For CBTs, there was a significant positive difference for responsiveness (Table 5), suggesting that CBTs perceived the anticoagulation service to significantly outperform their level of expectations with a positive gap score of 0.15 (p = 0.016).

Comparing the results in Tables 4 and 5 revealed that, when considering PSTMs, in all cases there were significant differences between Perceptions and Expectations. However, in the case of CBTs, there was no significant difference between Perceptions and Expectations, with the exception of responsiveness that was perceived to be delivering over and above the levels expected by this patient group. This indicates that, when compared with their Expectations, PSTMs perceive service quality aspects of the anticoagulation clinic relating to responsiveness as underperforming, whereas CBTs perceive over‐performance of the anticoagulation clinic relating to this construct.

### Choice

Finally, we explore the similarities and differences between CBTs and PSTMs with regard to the way in which they value having and making choices. The views of PSTMs and CBTs relating to having and making choices are summarised in Table 6. To ensure Cronbach's alpha consistency scores for the two scales would meet the necessary threshold, Items HC4 and MC4 were deleted for both PSTM and CBT responses.

**Table 6:** Having and making choices

Construct	Response options: (1) Strongly disagree to (7) Strongly agree	Cronbach's alpha		Mean (PSTM)	Mean (CBT)	Gap score (PSTM‐CBT)	Sig. (two‐tail)
		PSTM	CBT				
Having choices	HC1. I am the kind of person who likes to be offered choices rather than being told the best way forward	0.67 (Item HC4 deleted)	0.68 (Item HC4 deleted)	6.56	6.64	−0.08	0.418
	HC2. I prefer to know what options are available to me						
	HC3. I like to know all the possible ways in which I could be treated						
	HC4. I am not interested in finding out what all the options are for treating my problem^!^						
Making choices	MC1. I am happy for the doctor to make decisions for me^a^	0.69 (Item MC3 deleted)	0.66 (Item MC3 deleted)	4.94	4.08	0.86	0.000^b^
	MC2. It's not important to me to make my own healthcare decisions^a^						
	MC3. I prefer to make my own mind up about what treatment I will have						
	MC4. I am the kind of person who feels overwhelmed by choice and would rather it could be simpler^a^						

a Response options reversed to align semantics with other construct items, that is higher scores indicate positive responses to having choices or making choices.

b Significant at 99th percentile

In terms of the way in which having choices was valued, there was no significant difference between PSTMs and CBTs. Both groups agreed that it is important to have choices, with mean scores of 6.56 for PSTMs and 6.64 for CBTs. In respect of making choices, it is clear from the mean scores that making choices was valued less than having choices for both CBTs and PSTMs with mean scores of 4.94 for PSTMs and 4.08 for CBTs. The mean CBT score of 4.08 suggested that this patient group tended to have a neutral response to the statements, whilst PSTMs tended to slightly agree with the making choice statements (a value of 5 equated to a response of ‘slightly agree’ on the seven‐point Likert‐type scale for this instrument). There was, however, a significant difference between the scores of the two groups indicating that making choices is significantly more important for PSTMs compared with CBTs.

Finally, we sought to establish whether the difference between PSTMs and CBTs on the making choices variable might simply be a function of educational differences. Accordingly, we conducted an analysis of variance where making choices was the dependent variable with two independent variables: education (secondary vs. university vs. postgraduate) and group (PSTMs vs. CBTs). In line with Ogden et al. (2008), there was a main effect of education with those with higher level of education having a greater preference for making choices (*f* =* *8.06, df = 1, p = .000). *Post hoc* tests showed that the postgrad education group (mean = 5.28, p = .019) and the university education group (mean = 5.32, p = .002) both had a significantly greater preference for making choice than the secondary education group (mean = 4.26). However, importantly, there was also a main effect of group such that PSTMs had a greater preference for making choice than did CBTs (*f *= 7.49, df = 1, p = 0.007). There was no interaction effect.

## Discussion

This study investigated how two discrete patient groups enrolled as patients at the same anticoagulation clinic perceive service quality and value choice with a view to gaining insight into how the provision of clinical services may be adapted to better accommodate the needs of PSTMs with a view to improving levels of patient engagement in this emerging paradigm of care. In particular, this study considered those whose condition was monitored through the clinic and those whose monitoring was primarily conducted through their own STM practices. We considered and compared their perceptions and expectations of the clinical service quality provided (via the SERVQUAL instrument), and the extent to which they valued choice (via the ChQ instrument). In this section, the results are discussed in the context of the three key research questions and some resulting practice considerations for service provision proposed.

The first research question was to explore patient satisfaction levels using the SERVQUAL measures of Expectations and Perceptions, and the extent to which these differ between CBT and PSTM patient groups. Ratings of perceived service quality provided by their anticoagulation service showed satisfaction was generally high for both groups. However, CBTs perceived the service quality to be significantly higher compared with PSTM perceptions for all five SERVQUAL service quality construct measures. Given the quantitative nature and specific area of focus for this study, it is not possible to identify the specific reasons for the differences in respective SERVQUAL dimensions, particularly when considering individual CBT and PSTM patient perspectives of these differences. However, there may be some value in reflecting on what the key differences are in the provisions made by the anticoagulation service to facilitate patient engagement with each respective patient group, and to consider these differences in the light of existing research. One key difference in the way that the two groups engage with the clinic is that CBTs engage in regular face‐to‐face contact, whereas PSTMs engage in regular asynchronous telephone contact. Contact is considered to be asynchronous because PSTMs typically do not engage in real‐time communication with the clinic, but rather telephone messages are center on an answering machine by the patient, which are then responded to posting a dosage profile from the clinic to the patient's home. One possible factor that may have contributed to the service being perceived more positively by CBTs is the comparatively higher levels of face‐to‐face contact that this patient group has with practitioners, particularly for the responsiveness, assurance and empathy dimensions. Existing research has found that synchronous face‐to‐face communication between the patient and practitioner has been found to be a useful means of supporting patients and facilitating sustained engagement in positive health behaviours 73. Indeed, health communication research suggests that the direct and personalised interactions that occur within the privacy and trust of the patient–provider relationship have important influences on the perceived quality of care for both the patient and the practitioner, patient adherence to interventions and patient motivation 74, 75, 76, 77. There is also some existing evidence, specifically relating to chronic disease patients, which suggests that delivery of care purely via asynchronous means of communication (i.e. not real‐time communication) may lead to higher levels of patient disengagement 78. Another possible explanation could be that the PSTM mode of engagement does not dovetail with the services that have historically been designed to simply meet the needs of CBTs. In both cases, clinical services may benefit from identifying how existing aspects of service delivery could be adapted to improve how services are perceived by the PSTM patient group. Increasing the frequency of face‐to‐face contact with PSTMs may be counterproductive to the convenience and the service efficiencies afforded by the PSTM mode of engagement. However, some increased effort in developing communication modes that are considered as a more personalised and an effective proxy for this type of communication could possibly enhance satisfaction with the services provided by the clinic. Indeed, existing research suggests that patient satisfaction may be improved if patients are provided with a choice of modes of communication via which they may interact with clinical services 79. The first research question also considered *expected* satisfaction levels. In all cases, apart from tangibles, PSTMs and CBTs had the same levels of expectation of the service. This exception is perhaps not surprising given that PSTMs view and utilise the tangibles (i.e. the appearance of physical facilities, equipment, personnel and communication materials) much less frequently than CBTs and thus they are likely to be less salient aspects of service provision. However, patient expectations relating to interactions with clinical services (i.e. reliability, responsiveness, empathy) appear to be highly valued dimensions for both patient groups. From a service provider's perspective, this may be worth noting, particularly when considering changes to clinical service provision that necessitate less direct contact with particular patient groups. Prioritising the integration of these aspects of service delivery with new self‐care and management regimens is therefore likely to be a productive focus.

The second research question was to explore the relationship between the measures of expectations and perceptions for the CBT and PSTM patient groups, respectively. For all four SERVQUAL constructs considered, PSTMs perceptions of the service quality they received were significantly less positive than their expectations. CBTs overall perceptions were similar to expectations although for the responsiveness measure, a significant positive SERVQUAL gap score indicated that CBTs perceived the anticoagulation clinic to be outperforming expectations in terms of staff providing promptness of service, their willingness to help and prioritising provision of answers to patients' questions. This was thus the opposite pattern to PSTMs expectations and perceptions for responsiveness. This may perhaps be expected to be the case when considering that the interactions PSTMs have with the clinic often involve receiving a dosage profile by post a day or two after leaving a recorded telephone message of their INR self‐tested results, as opposed to CBTs, who receive their new dosing profile in person within minutes of the INR test is carried out. PSTMs do have the option of calling the clinic to speak directly with an anticoagulation specialist at any time; however, arguably, this may not relate directly to the concept of responsiveness. In considering how to embed the concept of service responsiveness with the service provision to PSTMs, the ‘over‐performance’ on this dimension for CBTs provides some encouragement for indicators of responsiveness that can be delivered by media that whilst in line with the more remote delivery model, which is core to STM, also communicate speedily to the receipt of self‐testing information by the clinic. For example, the clinic service could explore the possibility of immediately acknowledging receipt of patient self‐testing readings and sending dosage profiles via email and/or text messages which would be received more quickly and in a more personalised context by the patient. Additionally, these new communication platforms could be used to both receive and respond to any queries. These additional mechanisms for delivering ‘responsive’ communications could be perceived by patients as the clinic providing a wider range of communication platforms which demonstrate an increased willingness to help and prioritise provision of answers to patient questions. The extent to which these alternative methods of communication would be effective in meeting/maintaining PSTM expectations of responsiveness, however, remains to be seen. Future research is needed to explore the extent to which such new communication platforms may be deployed effectively.

The final research question was to explore the similarities and differences between CBTs and PSTMs with regard to the ways in which they value having and making choices. Results revealed that PSTMs and CBTs are both positive about having choices and attribute equal value to having choices about their care. Both PSTMs and CBTs were also less positive about the notion of making choices compared to having choices. These findings are in line with existing research findings on this topic 66, 67. However, a key finding was that there was a significant difference between PSTMs and CBTs in the extent to which they value *making* choices: PSTMs were significantly more positive about the notion of making choices than CBTs. This suggests that PSTMs appear to be more likely to subscribe to the model of the patient as the ‘consumer’, that is with a preference for making choices for themselves. On the other hand, CBTs appear to value having choices, but are less likely to be willing to make choices about their care and thus arguably have a preference for a more paternalistic approach to their care and the choices that are made 66. It is significant that this difference between the two groups was not a function of their level of education. It should be noted, however, that the majority of the participants (more than two‐thirds) were aged between 56 and 80 years. Therefore, this finding should not be taken to be representative of the normal population. It should also be noted that the anticoagulation service has a specific selection process by which patients become PSTMs, which may not be representative of other services. However, the selection process may provide some insights into the reasons for the differences found in this particular study between these two patient groups in their preferences for making choices. Existing CBTs that meet the self‐testing eligibility criteria are offered the chance to become a PSTM, and must then choose whether to engage with the self‐testing mode of care or remain as a CBT. Given that only a small proportion of those patients eligible to become PSTMs actually do so 21, it seems feasible that remaining as a clinic tester may perhaps simply represent a default continuance of the existing practice rather than an active choice to do so. On the other hand, PSTMs, who have a greater preference for making choice, have moved out of the default and made a decision to engage in the more active mode of care. Nevertheless, the finding that different patient groups have significantly different preferences with regard to making choices enables clinical services to tailor care pathways to better suit the preferences of each of the respective patient groups.

### Practice considerations

In summary, when considering the findings of this study, there are numerous practice considerations which service providers may benefit from paying attention to when designing and delivering clinical services to PSTMs. Taking into account these practice considerations may help to ensure that PSTM perceptions of a service match their expectations and better cater for their specific choice preferences.

1. Ensure that frequent, timely and personalised interactions are maintained with PSTMs.
2. Prioritise investment of resources to facilitate personalised patient/practitioner interaction over the investment in tangibles.
3. Maintain short response times between receiving self‐test results and communicating back the dosage profile to the patient.
4. Explore appropriate strategies that provide PSTMs with opportunities to make more choices about their care.
5. Explore ways in which it is appropriate to make self‐testing less a matter of active choice and a more standard method of the care pathway.

### Study limitations

This study was cross‐sectional in design, and hence, it is difficult to make causal inferences particularly about the relationship between PSTMs and their preferences for making choices. More specifically, it is not clear whether the preference for making choices was as a result of having become more partial to making choices as a function of having been a PSTM for some time, or whether this preference may have been as a result having made the choice to become a PSTM in the first place. Only a longitudinal study of CBTs and PSTMs may provide additional insight on the cause for the significant differences between these two patient groups. Furthermore, this study did not ask participants to report the length of time that they were CBTs, prior to becoming PSTMs. This additional information, if reported, may have provided additional insights into the profile of PSTMs that took part in this study and whether this factor had any significant relationship with their choice preferences.

## Conclusions

This study explored patient perceptions and expectations of service quality and how choice is valued by two discrete patient groups, PSTMs and CBTs. Perceptions and expectations of service quality were measured using the SERVQUAL instrument 49, and choice preferences were measured using the ChQ instrument 67. A between‐groups comparison revealed that CBTs perceived service quality to be significantly higher for all five SERVQUAL constructs compared with PSTMs. CBTs had significantly higher expectations of tangibles (i.e. the appearance of physical facilities, equipment and staff within the clinic) compared with PSTMs. A within‐groups comparison revealed that PSTMs perceptions of all SERVQUAL constructs considered were significantly less positive than their expectations. CBTs reported that the service they received exceeded their expectations for the SERVQUAL construct responsiveness. With regard to choice preferences, PSTMs considered making choices to be significantly more important than CBTs. The findings in this study provide valuable insights into the differences that exist in terms of how respective patient groups perceive services, what their expectations of a service are and the extent to which they value having and making choices about their care. If patients are to make the shift towards engaging in the STM care model, clinical services will benefit from strategically adapting their service provision to better cater for the needs of PSTMs and prioritise the design and delivery of services in line with their perceptions, expectations and choice preferences. Based on the outcomes of this study, a number of practice considerations have been identified which if taken into account may help to enable clinical services to more effectively cater for the needs of PSTMs with the goal of achieving higher levels of engagement with this mode of care delivery.

## Author contributions

All authors have participated in the design of the study and the preparation of the manuscript. Jasna Kuljis and Julie Barnett were the principal investigators with primary responsibility for the study. Arthur G. Money conducted all interviews and carried out analysis of data. Debbie Duffin and GD are anticoagulation specialist and is an provided clinical expertise for the results interpretation and formulating practice recommendations.

## Ethical approval

Ethical approval for this study was obtained via the Brunel University Ethical Approval Committee.

## Funding

This study was in part funded by grant number Ref: EP/G012393/1 from the Engineering and Physical Sciences Research Council. No conflict of interest has been declared by the author.

## References

[1] Darzi A , ed. High Quality Care for All: NHS Next Stage Review Final Report. 2008, Department of Health, London.

[2] Gilbert T , Powell J . Family, caring and ageing in the United Kingdom. Scand J Caring Sci 2005; 19: 53–7. 15737166 10.1111/j.1471-6712.2004.00313.x

[3] Money AG , Barnett J , Kuljis J , Lucas J . Patient perceptions of epinephrine auto‐injectors: exploring barriers to use. Scand J Caring Sci 2013; 27: 335–44. 22834703 10.1111/j.1471-6712.2012.01045.x

[4] Dale B , Söderhamn U , Söderhamn O . Self‐care ability among home‐dwelling older people in rural areas in southern Norway. Scand J Caring Sci 2012; 26: 113–22. 21883344 10.1111/j.1471-6712.2011.00917.x

[5] Swan M . Emerging patient‐driven health care models: an examination of health social networks, consumer personalized medicine and quantified self‐tracking. Int J Environ Res Public Health 2009; 6: 492–525. 19440396 10.3390/ijerph6020492 PMC2672358

[6] Heneghan C , Ward A , Perera R , Bankhead C , Fuller A , Stevens R , Bradford K , Tyndel S , Alonso‐Coello P , Ansell J , Beyth R , Bernardo A , Christensen TD , Cromheecke ME , Edson RG , Fitzmaurice D , Gadisseur AP , Garcia‐Alamino JM , Gardiner C , Hasenkam JM , Jacobson A , Kaatz S , Kamali F , Khan TI , Knight E , Körtke H , Levi M , Matchar D , Menéndez‐Jándula B , Rakovac I , Schaefer C , Siebenhofer A , Souto JC , Sunderji R , Gin K , Shalansky K , Völler H , Wagner O , Zittermann A . Self‐monitoring of oral anticoagulation: systematic review and meta‐analysis of individual patient data. Lancet 2012; 379: 322–34. 22137798 10.1016/S0140-6736(11)61294-4

[7] Shah SGS , Robinson I . patients' perspectives on self‐testing of oral anticoagulation therapy: content analysis of patients' internet blogs. BMC Health Serv Res 2011; 11: 25. 21291542 10.1186/1472-6963-11-25 PMC3045880

[8] Sigurdardottir AK , Jonsdottir H . Empowerment in diabetes care: towards measuring empowerment. Scand J Caring Sci 2008; 22: 284–91. 18298619 10.1111/j.1471-6712.2007.00506.x

[9] Robinson J , Callister LC , Berry JA , Dearing KA . Patient‐centred care and adherence: definitions and applications to improve outcomes. J Am Acad Nurse Pract 2008; 20: 600–7. 19120591 10.1111/j.1745-7599.2008.00360.x

[10] Barlow J , Wright C , Sheasby J , Turner A , Hainsworth J . Self‐management approaches for people with chronic conditions: a review. Patient Educ Couns 2002; 48: 177–87. 12401421 10.1016/s0738-3991(02)00032-0

[11] Goudswaard AN , Stolk RP , Zuithoff NP , de Valk HW , Rutten GE . Long‐term effects of self management education for patients with type 2 diabetes taking maximal oral hypoglycaemic therapy: a randomized trial in primary care. Diabet Med 2004; 21: 491–6. 15089797 10.1111/j.1464-5491.2004.01153.x

[12] Lip GY , Rudolf M , Kakar P . Management of atrial fibrillation: the NICE guidelines. Int J Clin Pract 2007; 61: 9–11. 17229173 10.1111/j.1742-1241.2006.01151.x

[13] DoH . Equity and Excellence: Liberating the NHS (White Paper). 2010, The Stationery Office, London, UK, 61.

[14] DoH . Liberating the NHS: No Decision about Me Without Me, Vol. 16942 2012, The Stationery Office, London, UK, 1–41.

[15] Kitchener T . Anticoagulation therapy. Nurs Stand 2011; 25: 59. 21329134 10.7748/ns2011.01.25.21.59.c8291

[16] Hibbard J , Mahoney ER , Stock R , Tusler M . Do increases in patient activation result in improved self‐management behaviours? Health Serv Res 2007; 42: 1443–63. 17610432 10.1111/j.1475-6773.2006.00669.x PMC1955271

[17] Anderson RM , Funnell MM . Patient empowerment: reflections on the challenge of fostering the adoption of a new paradigm. Patient Educ Couns 2005; 57: 153–7. 15911187 10.1016/j.pec.2004.05.008

[18] Kilo CM , Wasson JH . Practice redesign and the patient‐centred medical home: history, promises, and challenges. Health Aff 2010; 29: 773–8. 10.1377/hlthaff.2010.0012 20439860

[19] Greenhalgh T , Campbell‐Richards D , Vijayaraghavan S , Collard A , Malik F , Griffin M , Morris J , Claydon A , Macfarlane F . New models of self‐management education for minority ethnic groups: pilot randomized trial of a story‐sharing intervention. J Health Serv Res Policy 2011; 16: 28–36. 20739577 10.1258/jhsrp.2010.009159

[20] Yang DT , Robetorye RS , Rodgers GM . Home prothrombin time monitoring: a literature analysis. Am J Hematol 2004; 77: 177–86. 15389909 10.1002/ajh.20161

[21] Connock M . Clinical effectiveness and cost‐effectiveness of different models of managing long‐term oral anticoagulation therapy: a systematic review and economic modelling. Health Technol Assess 2007; 11: 1–86. 17903392 10.3310/hta11380

[22] Fitzmaurice DA , Gardiner C , Kitchen S , Mackie I , Murray ET , Machin SJ . An evidence‐based review and guidelines for patient self‐testing and management of oral anticoagulation. Br J Haematol 2005; 131: 156–65. 16197444 10.1111/j.1365-2141.2005.05739.x

[23] Quin J , Rogers LQ , Markwell S , Butler T III , McClafferty R , Hazelrigg S . Home anticoagulation testing: accuracy of patient reported values. J Surg Res 2007; 140: 189–93. 17397870 10.1016/j.jss.2007.01.036

[24] Matchar DB , Jacobson A , Dolor R , Edson R , Uyeda L , Phibbs CS , Vertrees JE , Shih MC , Holodniy M , Lavori P . Effect of home testing of international normalized ratio on clinical events. N Engl J Med 2010; 363: 1608–20. 20961244 10.1056/NEJMoa1002617

[25] Bloomfield HE , Krause A , Greer N , Taylor BC , MacDonald R , Rutks I , Reddy P , Wilt TJ . Meta‐analysis: effect of patient self‐testing and self‐management of long‐term anticoagulation on major clinical outcomes. Ann Intern Med, 2011; 154: 472–82. 21464349 10.7326/0003-4819-154-7-201104050-00005

[26] Garcia‐Alamino JM , Ward AM , Alonso‐Coello P , Perera R , Bankhead C , Fitzmaurice D , Heneghan CJ . Self‐monitoring and self‐management of oral anticoagulation. Cochrane Database Syst Rev 2010; 14: 4. 10.1002/14651858.CD003839.pub2 20393937

[27] Braun S , Spannagl M , Völler H . Patient self‐testing and self‐management of oral anticoagulation. Anal Bioanal Chem 2009; 393: 1463–71. 18568340 10.1007/s00216-008-2225-3

[28] Ansell J , Jacobson A , Levy J , Völler H , Hasenkam JM . Guidelines for implementation of patient self‐testing and patient self‐management of oral anticoagulation. Int J Cardiol 2005; 99: 37–45. 15721497 10.1016/j.ijcard.2003.11.008

[29] Gardiner C , Longair I , Pescott MA , Erwin H , Hills J , Machin SJ , Cohen H . Self‐monitoring of oral anticoagulation: does it work outside trial conditions? J Clin Pathol 2009; 62: 168–71. 19181634 10.1136/jcp.2008.059634 PMC2629005

[30] Wittkowsky AK , Sekreta CM , Nutescu EA , Ansell J . Barriers to patient self‐testing of prothrombin time: national survey of anticoagulation practitioners. Pharmacotherapy 2005; 25: 265–9. 15767240 10.1592/phco.25.2.265.56949

[31] Oertel LB , Libby EN . In patient self‐testing a good thing? J Thromb Thrombolysis 2010; 29: 214–8. 19902148 10.1007/s11239-009-0414-3

[32] Young C , Skorga P . Self‐monitoring and self‐management of oral anticoagulation. Int J Evid Based Med 2011; 9: 76–7. 10.1111/j.1744-1609.2010.00207.x 21332669

[33] Gardiner C , Williams K , Makie IJ . Patient self‐testing is a reliable and acceptable alternative to laboratory INR monitoring. Br J Haematol 2005; 128: 242–7. 15638860 10.1111/j.1365-2141.2004.05300.x

[34] Gardiner C , Williams K , Longair I , Mackie IJ , Machin SJ , Cohen H . A randomised control trial of patients self‐management of oral anti‐coagulation compared with patient self‐testing. Br J Haematol 2006; 132: 598–603. 16445833 10.1111/j.1365-2141.2005.05899.x

[35] Jordan JE , Osbourne RH . Chronic disease self‐management education programs: challenges ahead. Med J Aust 2007; 186: 84–7. 17223770 10.5694/j.1326-5377.2007.tb00807.x

[36] Keilman T , Huby G , Powell A , Sheikh A , Price D , Williams S , Pinnock H . From support to boundary: a qualitative study of the border between self‐care and professional care. Patient Educ Couns 2010; 79: 55–61. 19709844 10.1016/j.pec.2009.07.015

[37] Lawn S , Schoo A . Supporting self‐management of chronic health conditions: common approaches. Patient Educ Couns 2010; 80: 205–2011. 19931372 10.1016/j.pec.2009.10.006

[38] Pulvirenti M , McMillan J , Lawn S . Empowerment, patient centred care and self‐management. Health Expect, 2014; 17: 303–310. 22212306 10.1111/j.1369-7625.2011.00757.x PMC5060728

[39] Coster S , Norman I . Cochrane reviews of educational and self‐management interventions to guide nursing practice: a review. Int J Nurs Stud 2008; 46: 508–28. 19012889 10.1016/j.ijnurstu.2008.09.009

[40] Griffiths C , Foster G , Ramsay J , Eldridge S , Taylor S . How effective are expert patient (lay led) education programmes for chronic disease? Br Med J 2007; 334: 1254–6. 17569933 10.1136/bmj.39227.698785.47 PMC1892511

[41] Lawn S , McMillan J , Pulvirenti M . Chronic condition self‐management: expectations and responsibility. Patient Educ Couns 2011; 84: e5–e8. 20705412 10.1016/j.pec.2010.07.008

[42] Wilson PM , Kendall S , Brooks F . The expert patients programme: a paradox of patient empowerment and medical dominance. Health Soc Care Community 2007; 15: 426–38. 17685988 10.1111/j.1365-2524.2007.00701.x

[43] Wilson PM . The UK expert patients program: lessons learned and implications for cancer survivors' self‐care support programs. J Cancer Surviv 2008; 2: 45–52. 18648986 10.1007/s11764-007-0040-z

[44] Fox NJ , Ward KJ , O'Rourke AJ . The ‘expert patient’: empowerment or medical dominance? The case of weight loss, pharmaceutical drugs and the Internet. Soc Sci Med 2005; 60: 1299–309. 15626525 10.1016/j.socscimed.2004.07.005

[45] Greenhalgh T . Chronic illness: beyond the expert patient. Br Med J 2009; 338: 629–32. 10.1136/bmj.b49 19223339

[46] Scottish Executive Health Department . Our National Health: A Plan for Action, A Plan for Change. 2001, HMSO, Edinburgh.

[47] McWilliam CL . Patients, persons or partners? Involving those with chronic disease in their care. Chronic Illness 2009; 5: 277–92. 19933246 10.1177/1742395309349315

[48] Bury M . Researching patient‐professional interactions. J Health Serv Res Policy. 2004; 1: 48–54. 15006228 10.1258/135581904322724130

[49] Wisniewski M , Wisniewski W . Measuring service quality in a hospital colposcopy clinic. Int J Health Care Qual Assur 2005; 18: 217–28. 10.1108/09526860510594776 15974517

[50] Pakdil F , Harwood T . Patient satisfaction in a preoperative assessment clinic: an analysis using SERVQUAL dimensions. Total Qual Manag 2005; 16: 15–30.

[51] Duggirala M , Rajendran C , Anantharaman RN . Patient‐perceived dimensions of total quality service in healthcare. Benchmarking 2005; 15: 560–83.

[52] Snell L , White L , Dagger T . A socio‐cognitive approach to customer adherence in health care. Eur J Mark 2014; 48: 496–521.

[53] Gill L , White L . A critical review of patient satisfaction. Leadersh Health Serv 2009; 22: 8–19.

[54] Jordan JE , Briggs AM , Brand CA , Osborne RH . Enhancing patient engagement in chronic disease self‐management support initiatives in Australia: the need for an integrated approach. Med J Aust 2008; 189: 9. 19143585 10.5694/j.1326-5377.2008.tb02202.x

[55] Manary MP , Boulding W , Staelin R , Glickman SW . The patient experience and health outcomes. N Engl J Med 2013; 368: 201–3. 23268647 10.1056/NEJMp1211775

[56] Hudelson P , Cléopas A , Kolly V , Chopard P , Perneger T . What is quality and how is it achieved? Practitioners' views versus quality models. Qual Saf Health Care 2008; 17: 31–6. 18245217 10.1136/qshc.2006.021311

[57] Mosadeghrad AM . Healthcare service quality: towards a broad definition. Int J Health Care Qual Assur 2013; 26: 203–19. 23729125 10.1108/09526861311311409

[58] Kang G , James J . Service quality dimensions: an examination of Gronroo's service quality model. Manag Serv Qual 2004; 14: 266–77.

[59] Ladhari R . Alternative measures of service quality: a review. Manag Serv Qual 2008; 18: 65–86.

[60] Mosadeghrad AM . A conceptual framework for quality of care. Mater Sociomed 2012; 24: 251–61. 23922534 10.5455/msm.2012.24.251-261 PMC3732361

[61] Dagger TS , Sweeney JC , Johnson LW . A hierarchical model of health service quality. J Serv Res 2007; 10: 123–42.

[62] Sofaer S , Firminger K . Patient perceptions of the quality of health services. Annu Rev Public Health 2005; 26: 513–59. 15760300 10.1146/annurev.publhealth.25.050503.153958

[63] Kilbourne WE , Duffy J , Duffy M , Giarchi M . The applicability of SERVQUAL in cross‐national measurements of health‐care quality. J Serv Mark 2004; 18: 524–33.

[64] O'Connor S , Trinh H , Shewchuk RM . Perceptual gaps in understanding patient expectations for health care service quality. Health Care Manage Rev 2000; 25: 7–23. 10808414 10.1097/00004010-200004000-00002

[65] DoH . Choosing Health: Making Healthy Choices Easier. Vol. Cm 6374. 2004, The Stationery Office, London, UK, 207.

[66] Ogden J , Daniells E , Barnett J . The value of choice: the development of a new measurement tool. Br J Gen Pract 2008; 58: 614–8. 18801278 10.3399/bjgp08X330735 PMC2529198

[67] Barnett J , Ogden J , Daniels E . The value of choice: a qualitative study. Br J Gen Pract 2008; 58: 609–13. 18801277 10.3399/bjgp08X330717 PMC2529197

[68] Barry MJ , Edgman‐Levitan S . Shared decision making: the pinnacle of patient‐centered care. N Engl J Med 2012; 366: 780–1. 22375967 10.1056/NEJMp1109283

[69] Coulter A . Paternalism or partnership? Patients have grown up – and there's no going back. Br Med J 1999; 319: 719–20. 10487980 10.1136/bmj.319.7212.719 PMC1116580

[70] Anderson EA . Measuring service quality at a university health clinic. Int J Health Care Qual Assur 1995; 8: 32–37. 10142015 10.1108/09526869510081866

[71] Zeithaml VA , Parasuraman A , Berry LL . Delivering Quality Service: Balancing Customer Perceptions and Expectations. 1990, The Free Press, New York, USA.

[72] Hair JF , Black W , Babin B , Anderson R . Multivariate Data Analysis, 6th edn 2006, Prentice Hall, Upper Saddle River, NJ, USA.

[73] Wanyonyi KL , Themessl‐Huber M , Humphris G , Freeman R . A systematic review and meta‐analysis of face‐to‐face communication of tailored health messages: implications for practice. Patient Educ Couns 2011; 85: 348–55. 21397434 10.1016/j.pec.2011.02.006

[74] Heritage J , Maynard DW . Communication in Medical Care: Interaction Between Primary Care Physicians and Patients. 2006, Cambridge University Press, Cambridge.

[75] Francis LP . The physician‐patient relationship and a national health information network. J Law Med Ethics 2010; 38: 36–49. 20446982 10.1111/j.1748-720X.2010.00464.x

[76] Adler R , Vasiliadis A , Bickell N . The relationship between continuity and patient satisfaction: a systematic review. Fam Pract 2010; 27: 171–8. 20053674 10.1093/fampra/cmp099

[77] Mohr DC , Cuijpers P , Lehman K . Supportive accountability: a model for providing human support to enhance adherence to eHealth interventions. J Med Internet Res 2011; 13: e30. 21393123 10.2196/jmir.1602 PMC3221353

[78] Rosser BA , Vowles KE , Keogh E , Eccleston C , Mountain GA . Technologically assisted behaviour change: a systematic review of studies of novel technologies for the management of chronic illness. J Telemed Telecare 2009; 15: 327–38. 19815901 10.1258/jtt.2009.090116

[79] McGeady D , Kujala J , Ilvonen K . The impact of patient–physician web messaging on healthcare service provision. Int J Med Inform 2008; 77: 17–23. 17188564 10.1016/j.ijmedinf.2006.11.004

